# Electroreductive coupling of benzaldehyde by balancing the formation and dimerization of the ketyl intermediate

**DOI:** 10.1038/s41467-022-35463-3

**Published:** 2022-12-23

**Authors:** Jia Yu, Peng Zhang, Lulu Li, Kailang Li, Gong Zhang, Jia Liu, Tuo Wang, Zhi-Jian Zhao, Jinlong Gong

**Affiliations:** 1 School of Chemical Engineering and Technology, Key Laboratory for Green Chemical Technology of Ministry of Education, Tianjin University, 300072 Tianjin, China; 2 Collaborative Innovation Center of Chemical Science and Engineering (Tianjin), 300072 Tianjin, China; 3 Haihe Laboratory of Sustainable Chemical Transformations, 300192 Tianjin, China; 4 Joint School of National University of Singapore and Tianjin University, International Campus of Tianjin University, Binhai New City, 350207 Fuzhou, China

**Keywords:** Heterogeneous catalysis, Electrocatalysis, Electrocatalysis

## Abstract

Electroreductive coupling of biomass-derived benzaldehyde offers a sustainable approach to producing value-added hydrobenzoin. The low efficiency of the reaction mainly ascribes to the mismatch of initial formation and subsequent dimerization of ketyl intermediates (Ph-CH = O → Ph-C·-OH → Ph-C(OH)-C(OH)-Ph). This paper describes a strategy to balance the active sites for the generation and dimerization of ketyl intermediates by constructing bimetallic Pd/Cu electrocatalysts with tunable surface coverage of Pd. A Faradaic efficiency of 63.2% and a hydrobenzoin production rate of up to 1.27 mmol mg^−1^ h^−1^ (0.43 mmol cm^−2^ h^−1^) are achieved at −0.40 V *vs*. reversible hydrogen electrode. Experimental results and theoretical calculations reveal that Pd promotes the generation of the ketyl intermediate, and Cu enhances their dimerization. Moreover, the balance between these two sites facilitates the coupling of benzaldehyde towards hydrobenzoin. This work offers a rational strategy to design efficient electrocatalysts for complex reactions through the optimization of specified active sites for different reaction steps.

## Abstract (web summary)

Electroreductive coupling of benzaldehyde is mainly hindered by the mismatch between initial formation and subsequent dimerization of the ketyl intermediates. Here the authors report a strategy to balance the active sites for the generation and dimerization of the ketyl intermediates, leading to promoted production rate of hydrobenzoin.

## Introduction

Biomass-derived aromatic aldehydes and ketones are promising alternatives to petroleum as industrial feedstocks, among which benzaldehyde and its derivatives are important representatives^1–6^. The reductive coupling of benzaldehyde can generate value-added hydrobenzoin as a structural motif in antiepileptic drugs (e.g., Phenytoin Sodium) and photo-initiators^7,8^. In the traditional thermocatalytic coupling approach for hydrobenzoin production, low-valent metal reducing reagents and harsh reaction conditions are needed^9,10^. In contrast, electroreductive coupling of benzaldehyde with electrons as the reductive reagents can be carried out at room temperature and mild pressure, showing the potential to realize mass production of hydrobenzoin in a more sustainable manner^11–14^.

Ketyl intermediates (Ph-C·-OH) has been proved to be the key intermediates in electroreductive coupling of aldehydes^15,16^. The performance of an electrocatalyst for the coupling of aldehydes is determined by its ability to simultaneously generate and dimerize the ketyl intermediate. Recently, molecular^17^, carbon-based^18–20^ and metallic^18,21–23^ catalysts have been applied to promote the reductive coupling reactions. Molecular and carbon-based catalysts exhibit considerable dimerization selectivity^17–20^. However, they are less active to convert aldehydes into corresponding ketyl intermediates, resulting in high overpotential and unsatisfactory production rate^20,22^. Similarly, Cu, as a promising metallic electrocatalyst, shows relatively good capability to enhance the coupling step at a high conversion rate, but suffers from inadequate ability to active benzaldehyde^15,21^. A low ketyl intermediate concentration on the surface is likely the primary reason for the limited Faradaic efficiency (FE) for dimerization products on Cu^22^. On the contrary, Pd has preferable benzaldehyde adsorption and activation ability^24^. However, Pd predominately promotes the generation of benzyl alcohol instead of hydrobenzoin for the lacking of coupling sites in the electrocatalytic process^6,25^. Therefore, equilibrium between the active sites for the formation and dimerization of the ketyl intermediate is of great significance for achieving a considerable reaction rate of the electroreductive coupling reaction.

This paper describes the design and fabrication of a series of Pd/Cu catalysts with tunable Pd coverage to balance the active sites for the formation and dimerization of ketyl intermediate. The Pd/Cu catalyst with an optimized Pd coverage exhibits the highest coupling selectivity with a FE of 63.2% and a yield of 85.3%. The production rate of hydrobenzoin is up to 1.27 mmol mg^−1^ h^−1^ (0.43 mmol cm^−2^ h^−1^), which is higher than most previously reported catalysts. Revealed by density functional theory (DFT) calculations and spectroscopy experiments, Pd provides the generation sites and Cu acts as the dimerization sites for the ketyl intermediate. The balance between the formation and dimerization sites for ketyl intermediate on bimetallic Pd/Cu catalysts contributes to the enhanced FE and production rate for hydrobenzoin.

## Results

### Fabrication and characterization

**Fig. 1:**
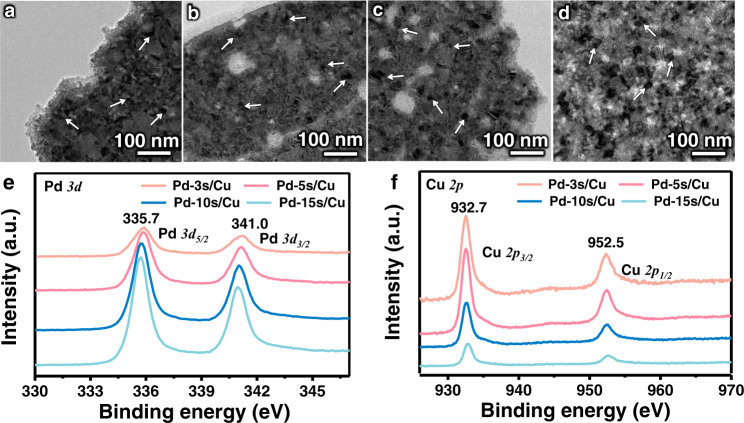
Characterization of Pd/Cu catalysts. **a**–**d** TEM images of Pd-3s/Cu (**a**), Pd-5s/Cu (**b**), Pd-10s/Cu (**c**) and Pd-15s/Cu (**d**). **e**, **f** Pd *3d* (**e**) and Cu *2p* (**f**) XPS spectra of as-prepared Pd/Cu catalysts.

The bimetallic Pd/Cu electrocatalysts were fabricated through sequential sputtering of Cu and Pd (experimental details can be found in the Methods). By adjusting the sputtering time of Pd from 3 to 15 s, the relative coverage between Pd and Cu at the surface can be well controlled. The obtained electrocatalysts are denoted as Pd-3s/Cu, Pd-5s/Cu, Pd-10s/Cu, and Pd-15s/Cu, with the suffix of “Pd” indicating its sputtering time. Monometallic Cu and Pd control samples were also fabricated through similar procedures. Scanning electron microscopy (SEM) characterization confirms these Pd/Cu catalysts exhibited porous structures (Supplementary Fig. 1). Transmission electron microscopy (TEM) images show the uniform presence of small Pd particles with sizes of around 10 nm on the Cu (Fig. 1a–d and Supplementary Fig. 2). High-resolution TEM (HRTEM) observation of Pd-5s/Cu (Supplementary Fig. 3) further confirms the decoration of Pd particles (with the lattice spacing of 0.22 nm for the Pd(111) facet) on Cu surface (with the lattice spacing of 0.20 nm for the Cu(111) facet). In the Pd/Cu catalysts, only the diffraction peaks of Cu are observed, which could be attributed to the small particle size and low content of Pd (Supplementary Fig. 4). High-resolution X-ray photoelectron spectroscopy (XPS) characterization was carried out to reveal the varied surface atomic ratio of Pd in the different Pd/Cu samples^26,27^ (Fig. 1e, f). The surface atomic ratio of Pd/Cu increases almost linearly with the sputtering time of Pd (Supplementary Fig. 5)^27,28^. This result indicates the relative ratio between Pd and Cu sites at the surface can be well tuned at a certain range.

### Performance evaluation

**Fig. 2:**
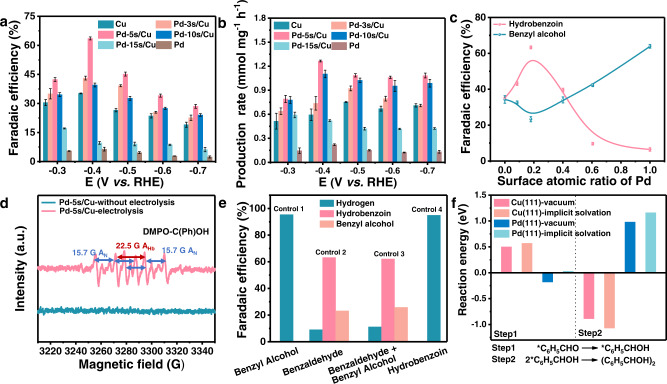
Catalytic performance of Pd/Cu catalysts and results of DFT calculations. **a** Faradaic efficiencies and (**b**) average production rates of hydrobenzoin at different potentials. **c** Faradaic efficiencies of hydrobenzoin (pink line) and benzyl alcohol (green line) at −0.40 V vs. RHE as a function of surface atomic ratio of Pd. **d** EPR spectra over Pd-5s/Cu with and without electrolysis. **e** Faradaic efficiencies of different products for control experiments on Pd-5s/Cu. **f** Calculated reaction energy of the formation and dimerization of ketyl intermediate in benzaldehyde reduction using computational hydrogen electrode model. Error bars represent the standard deviation from at least three independent measurements.

The performance of Pd/Cu for electroreductive coupling of benzaldehyde was evaluated in a three-electrode H-type cell, with 40 mmol L^−1^ benzaldehyde in 0.1 M KOH as the electrolyte. The liquid and gaseous products were quantified by liquid chromatography (LC) and gas chromatography (GC), respectively. The formation of hydrobenzoin was further ensured by mass spectrometry (MS) and ^1^H nuclear magnetic resonance (NMR) spectroscopy (Supplementary Figs. 6, 7). The carbon balance is nearly 100% in all the benzaldehyde electrolysis reactions (Supplementary Fig. 8). The introduction of Pd did not influence the ECSA of Pd/Cu catalysts significantly (Supplementary Fig. 9, 10). With the increase of surface Pd content, the FE for hydrobenzoin shows a volcano trend (Fig. 2a and Supplementary Fig. 11). The highest FEs at different potentials are all achieved by Pd-5s/Cu. Similar trends could also be found in the production rates and yields of hydrobenzoin by different samples at varied potentials (Fig. 2b and Supplementary Fig. 12). The highest FE for hydrobenzoin of 63.2% is achieved by Pd-5s/Cu at −0.40 V vs. reversible hydrogen electrode (RHE) with the yield of 85.3% and the production rate of 1.27 mmol mg^−1^ h^−1^ (0.43 mmol cm^−2^ h^−1^), which compares favorably to most reported catalysts^15,16,21,23,29–32^ (Supplementary Table 1 and Supplementary Fig. 13, 14). The stability of Pd-5s/Cu was also evaluated. Its good performance remains stable after 10 cycling tests with almost constant FE for hydrobenzoin (Supplementary Fig. 15). The porous morphology and the main crystal phase of Pd-5s/Cu are nearly unchanged (Supplementary Figs. 16, 17).

Further analyses of the catalytic performance show that the FE for benzyl alcohol first decreases and then increases with the enlargement of Pd coverage, reaching the minimum on Pd-5s/Cu (Fig. 2c and Supplementary Fig. 18). It is worth noting that the main organic product on Pd is benzyl alcohol (Supplementary Fig. 11f). This result indicates that a large amount of ketyl intermediate might be formed on Pd, which can only be further converted to benzyl alcohol due to the poor active of Pd to catalyze the dimerization reaction^6^. The poor catalytic performance of Pd towards the dimerization reaction can also be reflected by the consistently low FEs for hydrobenzoin at different potentials (Supplementary Fig. 11f). In contrast to the potential-dependent activity of Pd to generate benzyl alcohol, the FE of Cu for benzyl alcohol seems to be not apparently affected by the applied potential (Supplementary Fig. 18). Such phenomenon could possibly be explained by the poor interaction with benzaldehyde and the relatively low activity to activate benzaldehyde into the ketyl intermediate on Cu^15,22^. A limited amount of ketyl intermediate on the Cu might lead to the restricted and potential-independent selectivity towards benzyl alcohol. Therefore, increasing the coverage of Pd on Cu could promote the generation of the ketyl intermediate, which provides sufficient reactant for the coupling reaction on Cu to produce hydrobenzoin (Fig. 2c). However, after reaching the optimized surface coverage by Pd-5s/Cu, further enlargement of Pd coverage may induce excess ketyl intermediate and shrink the surface Cu sites for the coupling reaction. As a consequence, the FE for the hydrobenzoin decreases with the promoted generation of benzyl alcohol. Thus, balancing the surface Pd active sites for the generation and Cu active sites for the dimerization of the ketyl intermediate could be significant to improve the FE for the electroreductive coupling reaction. To support this hypothesis, it is essential to clarify the important role of the ketyl intermediate for the electroreduction reaction and specific catalytic functions of the Pd and Cu.

### Mechanism insight

Electron paramagnetic resonance (EPR) spectroscopy characterization and control experiments were carried out to illustrate the importance of the ketyl intermediate to support the proposed mechanism. Quasi in situ EPR experiments were performed with 5,5-dimethyl-1-pyrroline-N-oxide (DMPO) as the trapping agent. After electrolysis with the Pd-5s/Cu electrode at −0.40 V vs. RHE for 8 min, the sample was taken by capillary tubes for EPR experiments. The signal for carbon radicals was detected as a sextet (Fig. 2d), which confirms the formation of the ketyl intermediate. However, no signal was observed without electrolysis, revealing that the ketyl intermediate is formed during the electrochemical process^18,33^. As for the control experiment, when anhydrous acetonitrile, an aprotic solvent, was used as the electrolyte, barely any product was obtained. This result indicates that the formation of the ketyl intermediate, which consumes the source of H^+^ derived from H_2_O dissociation, is essential. In benzyl alcohol reduction experiment, only H_2_ could be obtained in 40 mmol L^-1^ benzyl alcohol solution (Fig. 2e), indicating benzyl alcohol is not the intermediate for the formation of hydrobenzoin. When benzyl alcohol is added to the benzaldehyde electrolyte for the performance evaluation, the FE for hydrobenzoin remains constant, suggesting the hydrobenzoin is not obtained by the coupling between benzyl alcohol and benzaldehyde. The electrolysis was also carried out in hydrobenzoin solution with H_2_ as the only product, demonstrating that the benzyl alcohol byproduct would only be generated by the further reduction of the ketyl intermediate. All these results indicate the ketyl intermediate could be the key intermediate in the production of hydrobenzoin.

To clarify the corresponding roles of Pd and Cu in this reaction, cyclic voltammograms (CV) experiments, Tafel analysis, and theoretical calculations were carried out. CV curves in different electrolytes could provide information about the catalytic properties of electrodes by tracking the processes involving charge transfer^21^. No shallow peak was observed in the CV curves of Cu and Pd in 0.1 M KOH (Supplementary Fig. 19). When 40 mmol L^−1^ benzaldehyde was induced in the 0.1 M KOH electrolyte, a current peak at −0.50 V vs. RHE appeared on Cu, suggesting the activation of benzaldehyde (Supplementary Fig. 20a). As for Pd, this current peak was observed at −0.40 V vs. RHE, nearly 0.10 V more positive than that on Cu (Supplementary Fig. 20b), indicating the enhanced benzaldehyde activation ability of Pd^5,34^. To corroborate better benzaldehyde activation on Pd in terms of reaction energy, the density functional theory (DFT) calculations were carried out on Cu(111) and Pd(111) (Supplementary Fig. 21, 22), which have been considered as the stable facets for catalysts fabricated through the sputtering approach^35^. The reaction barrier for the generation of ketyl intermediate on Pd was lower than on Cu according to the simulation results (Fig. 2f). Moreover, the Pd showed a lower Tafel slop than Cu, implying Pd exhibits a better benzaldehyde activation ability^36–38^ (Supplementary Fig. 23). These results indicated Pd can promote the activation of benzaldehyde. Moreover, the calculated benzaldehyde binding energy is lower on Pd than that on Cu, suggesting a stronger benzaldehyde adsorption ability on Pd (Supplementary Fig. 24). On the other hand, the reaction energy of the dimerization step on Cu is calculated to be −0.89 eV, much lower than the barrier on Pd (0.98 eV), implying a predominant role of Cu for the coupling step (Fig. 2f). Thus, Cu is suggested to be the main coupling site in Pd/Cu catalyst^15^, which could also be reflected by the results of the performance tests. Moreover, the effect of the presence of liquid phase and potential on the reaction was also considered (Supplementary Table 2). With the presence of liquid and potential, the difference in the values of binding and reaction energies is quite small and the reaction trends are consistent. According to these experimental results and theoretical calculations, the introduction of Pd could help to afford more initial ketyl intermediate and Cu could act as the subsequent dimerization sites on Pd/Cu catalysts. Therefore, it is proposed that balancing the two types of active sites would lead to promoted production of hydrobenzoin.

**Fig. 3:**
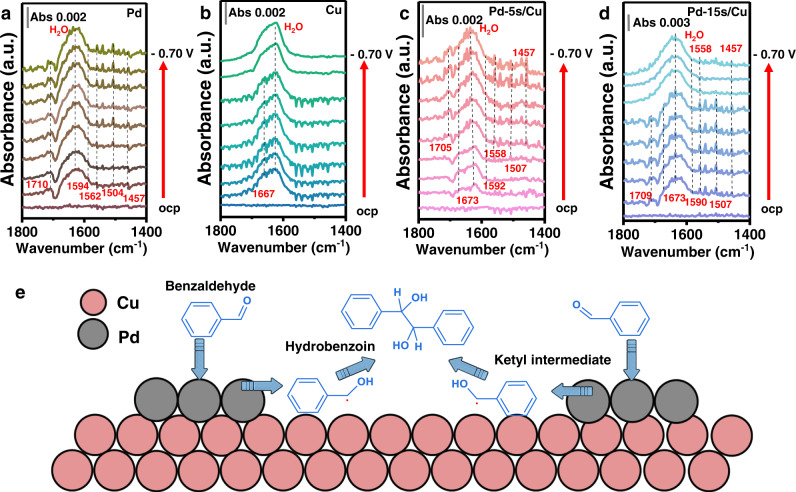
In situ ATR-SEIRAS investigation of Pd/Cu catalysts. **a**–**d** In situ ATR-SEIRAS spectra of Pd (**a**), Cu (**b**), Pd-5s/Cu (**c**), and Pd-15s/Cu (**d**). **e** Proposed reaction mechanism for the electroreductive coupling of benzaldehyde to hydrobenzoin.

To understand the significance of the optimization of active sites for the formation and dimerization of ketyl intermediate, spectroscopic studies were carried out. The in situ attenuated total reflectance-surface-enhanced infrared absorption spectroscopy (ATR-SEIRAS) was detected in a H-cell. The absorption band at ca. 1640 cm^−1^ is attributed to the H–O–H bending vibration of H_2_O^39,40^ (Supplementary Fig. 25). The vibration bands range from 1457 cm^−1^ to 1594 cm^−1^ could be attributed to the C-C stretching of the phenyl ring^41^. These adsorption bands were detected on Pd and Pd/Cu catalysts, and were absent on Cu (Fig. 3a–d). The vibrational bands at *ca*. 1710 cm^−1^ correspond to the C = O stretching mode of the benzaldehyde carbonyl group. This peak can be observed on Pd at all applied potentials^15,21^ (Fig. 3a). As for Cu, C = O stretching was also absent (Fig. 3b). These results indicate weaker benzaldehyde adsorption on Cu compared with Pd. On Pd-5s/Cu, the C = O stretching band locating at *ca*. 1705 cm^−1^ can be observed at −0.30 to −0.70 V vs. RHE (Fig. 3c). On Pd-15s/Cu, the C = O stretching shifts to *ca*. 1709 cm^−1^ at relatively lower potentials ranging from 0 to −0.40 V vs. RHE (Fig. 3d). Such phenomena imply enhanced benzaldehyde activation with the enlargement of Pd coverage^42–44^. Thus, the introduction of Pd would help to enhance the adsorption and activation of benzaldehyde on Pd/Cu catalysts^22,23^. The stretching band at *ca*. 1673 cm^−1^ could be assigned to the C–O vibration of the ketyl intermediate, which is the key intermediate of dimerization products^15^. The C–O vibration of the ketyl intermediate is absent on Pd, in accordance with its poor benzaldehyde coupling performance^15^. The C–O vibration of the ketyl intermediate on Cu appears at 0 V vs. RHE and disappears at −0.50 V vs. RHE. The C–O vibration of ketyl intermediate on Pd-5s/Cu is observed at 0 to −0.70 V vs. RHE, indicating stabilized ketyl intermediate at more negative potentials with moderate content of Pd^36,45^. As for Pd-15s/Cu, this peak appears at 0 V vs. RHE and disappears at −0.40 V vs. RHE. The disappearance of C–O vibration after −0.40 V vs. RHE might be ascribed to the rapid conversion of the ketyl intermediate to benzyl alcohol due to the shortage of coupling sites and the high active of Pd to promote the ketyl intermediate reduction reaction. According to these spectroscopy results, balancing the Pd sites for the formation and Cu sites for the dimerization of ketyl intermediates should be essential for realizing the high generation rate of hydrobenzoin (Fig. 3e).

**Fig. 4:**
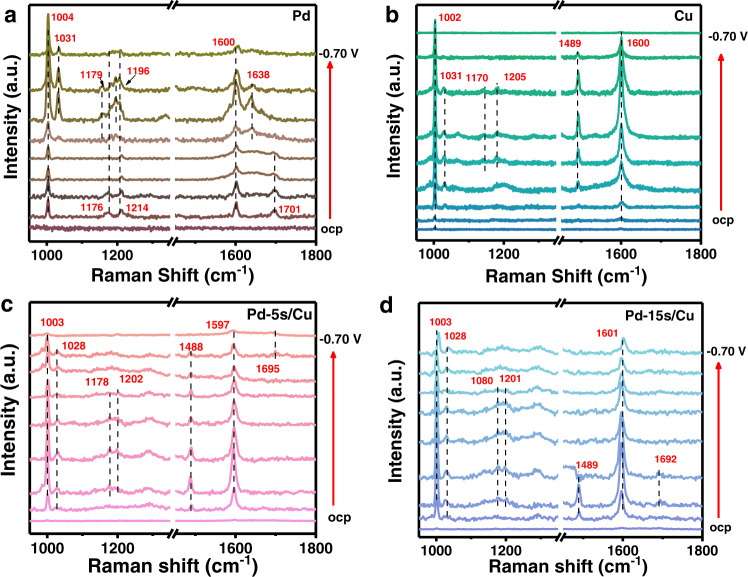
Raman spectroscopic investigation of Pd/Cu catalysts. In situ Raman spectra of benzaldehyde electroreduction over **a** Pd, **b** Cu, **c** Pd-5s/Cu, and **d** Pd-15s/Cu in 0.1 M KOH electrolyte with 40 mmol L^−1^ benzaldehyde.

In addition, in situ Raman spectra were also collected to further support the result of the ATR-SEIRAS study. Before the in situ experiments, the Raman spectra of the reactants, products, and benzoic acid from possible contamination were recorded to ensure the appropriate peak assignment in the experiments (Supplementary Fig. 26). Moreover, the Raman spectra in 0.1 M KOH on Pd-5s/Cu was also detected to rule out the adsorption species in blank solution (Supplementary Fig. 27). In Fig. 4, the ring-breathing mode of benzaldehyde appears at about 1003 cm^−1^ ^5^. The C = O stretching of benzaldehyde at *ca*. 1700 cm^−1^ is observed on Pd and Pd/Cu catalysts. However, it was absent on Cu, suggesting weaker benzaldehyde compared with Pd, in accordance with the ATR-SEIRAS results and the DFT calculations^18^. This deduction could be further supported by the observation of vibration bands at *ca*. 1174–1214 cm^−1^, assigned to the aromatic C − H bending of the benzene ring, on Pd at all applied potentials^4,41^ (Fig. 4a). These C−H bending peaks only appear at −0.30 to −0.50 V vs. RHE for Cu, showing the relatively poor performance of Cu for the activation of benzaldehyde (Fig. 4b). Such vibration bands of benzene ring are observed at wider potentials on Pd-5s/Cu and Pd-15s/Cu (Fig. 4c-d), which implies enhanced benzaldehyde adsorption with the enlargement of Pd coverage. The vibration band at *ca*. 1488 cm^−1^, which was not found in reactants or products, might be attributed to the ring stretching mode of ketyl intermediate^15^. This peak was not observed on Pd, probably attributing to the rapid conversion of ketyl intermediate to benzyl alcohol. The ring stretching mode of the ketyl intermediate appears on Cu at −0.20 V vs. RHE and disappears at −0.60 V vs. RHE. On Pd-5s/Cu, this peak was observed from 0 V to −0.60 V vs. RHE, indicating a stabilized ketyl intermediate at a wider potential range, which is in accordance with its maximum FE for hydrobenzoin^46^. However, this peak disappears after −0.20 V vs. RHE on Pd-15s/Cu. The rapid disappearance of this peak may be ascribed to the conversion of ketyl intermediate to benzyl alcohol in the cases of unmatched dimerization sites, as reflected by the high FE for benzyl alcohol of Pd-15s/Cu. Thus, these Raman results complement the ATR-SEIRAS experiments, and further illustrate the proposed reaction process, in which Pd generates the ketyl intermediates and Cu enhances their dimerization towards hydrobenzoin. The balancing of the two sites could be important for the design of high-performance electrocatalyst.

## Discussion

In conclusion, an effective approach to balancing active sites for the formation and dimerization of the ketyl intermediate was proposed to realize the highly efficient electroreductive coupling of benzaldehyde towards hydrobenzoin. With the optimized active sites, the Pd-5s/Cu achieved a FE of 63.2% and a yield of 85.3% with the hydrobenzoin production rate of 1.27 mmol mg^−1^ h^−1^ (0.43 mmol cm^−2^ h^−1^) at −0.40 V vs. RHE. In situ benzaldehyde adsorption experiments, Tafel analysis and DFT calculations reveal that the Pd sites are responsible for the activation of the ketyl intermediate and Cu provides sites for the subsequent dimerization reaction in the bimetallic electrocatalysts. Spectroscopy studies confirm the significance of balancing these active sites to promote the reaction. This work provides insights into the mechanism of the electrocatalytic reductive coupling of benzaldehyde. The findings of the work would guide an avenue toward the design of functional catalysts for the efficient electrosynthesis of value-added products.

## Methods

### Preparation of Cu, Pd/Cu, and Pd electrodes

Cu, Pd/Cu and Pd electrodes were prepared by controlled sputtering of Cu and Pd on carbon paper in a custom-made direct current (DC) magnetron sputtering system. The chamber pressure was maintained at 10^−5^ Pa before sputtering. During the deposition process, Ar was continuously introduced into the chamber with a flow rate of 20 sccm. The Cu electrode was prepared by sputtering Cu for 7 minutes. The Pd electrode was prepared by sputtering Pd for 10 minutes. The Pd-3s/Cu, Pd-5s/Cu, Pd-10s/Cu and Pd-15s/Cu were prepared by initial sputtering of Cu for 7 minutes and subsequent sputtering of Pd for 3 s, 5 s, 10 s, and 15 s, respectively.

### Activity evaluation

All the tests were carried out in an H-type cell (Supplementary Fig. 28). The anolyte (0.1 M KOH) and the catholyte (0.1 M KOH electrolyte with 40 mmol L^−1^ benzaldehyde) were separated by a bipolar membrane (BPM, Fumasep FBM-PK, Fumatech). The catholyte was stirred for 10 min with Ar protection before electrolysis. Carbon rod and Ag/AgCl (saturated KCl) electrodes were used as the anode and the reference, respectively. The carbon paper of the working electrode was cut into the size of 2.5 cm^2^, and the geometric surface area for activity tests is 2 cm^2^. The back side of the carbon paper is covered with Kapton. All the potentials in this paper were converted to the reversible hydrogen electrode (RHE) through the following equation:

1 $${{{{{\rm{E}}}}}}\,({{{{{\rm{RHE}}}}}})={{{{{\rm{E}}}}}}({{{{{\rm{Ag}}}}}}/{{{{{\rm{AgCl}}}}}})\,+\,0.197\,+\,0.059\,{{{{{\rm{pH}}}}}}$$

During the reaction, the catholyte was continually stirred at ~900 rpm with Ar as the protective gas and carrier gas (flow rate: 20 sccm). The pH of the electrolyte was monitored before (13.1) and after (13.2) the reaction to make sure no obvious change was observed. The products were quantified after the amount of electron flowing through the cathode achieved 100 C as controlled by a potentiostat (Autolab PGSTAT204, Metrohm). Gaseous products were analyzed with an online gas chromatography (GC7890B, Agilent Technologies, Inc.). All liquid products were collected and diluted twofold and analyzed by high-performance liquid chromatography (HPLC, Agilent Technologies 120 Infinity) equipped with a variable wavelength detector (VWD). The products were analyzed with a C18 column. The mobile phase is methanol/5% ammonium formate (V/V = 6:4) solution with a flow rate of 1 mL min^−1^. The carbon balance, FE, and production rate are calculated according to Eqs. (2)–(7):

2 $${{{{{\rm{Carbon}}}}}}\,{{{{{\rm{balance}}}}}}=\frac{{{{{{{\rm{n}}}}}}}_{{{{{{\rm{benzaldehyde}}}}}}}+{{{{{{\rm{n}}}}}}}_{{{{{{\rm{benzyl}}}}}}\,{{{{{\rm{alcohol}}}}}}}+2{{{{{{\rm{n}}}}}}}_{{{{{{\rm{hydrobenzoin}}}}}}}}{{{{{{{\rm{n}}}}}}}_{{{{{{\rm{benzaldehyde}}}}}}}^{0}}\times 100\%$$

3 $${{{{{{\rm{FE}}}}}}}_{{{{{{\rm{H2}}}}}}}=\frac{{{{{{{\rm{2n}}}}}}}_{{{{{{\rm{H2}}}}}}}\times {{{{{\rm{F}}}}}}}{{{{{{\rm{Q}}}}}}}\times 100\%$$

4 $${{{{{{\rm{FE}}}}}}}_{{{{{{\rm{hydrobenzoin}}}}}}}=\frac{{{{{{{\rm{2n}}}}}}}_{{{{{{\rm{hydrobenzoin}}}}}}}\times {{{{{\rm{F}}}}}}}{{{{{{\rm{Q}}}}}}}\times 100\%$$

5 $${{{{{{\rm{FE}}}}}}}_{{{{{{\rm{benzyl}}}}}}\,{{{{{\rm{alcohol}}}}}}}=\frac{{{{{{{\rm{2n}}}}}}}_{{{{{{\rm{benzyl}}}}}}\,{{{{{\rm{alcohol}}}}}}}\times {{{{{\rm{F}}}}}}}{{{{{\rm{{Q}}}}}}}\times 100\%$$

6 $${{{{{\rm{Production}}}}}}\,{{{{{{\rm{rate}}}}}}}_{{{{{{\rm{hydrobenzoin}}}}}}}({{{{{\rm{mmol}}}}}}\,{{{{{{\rm{mg}}}}}}}^{-1}{{{{{{\rm{h}}}}}}}^{-1})=\frac{{{{{{{\rm{n}}}}}}}_{{{{{{\rm{hydrobenzoin}}}}}}}}{{{{{{\rm{M}}}}}}\times {{{{{\rm{t}}}}}}}\times 100\%$$

7 $${{{{{\rm{Production}}}}}}\,{{{{{{\rm{rate}}}}}}}_{{{{{{\rm{hydrobenzoin}}}}}}}({{{{{\rm{mmol}}}}}}\,{{{{{{\rm{cm}}}}}}}^{-2}{{{{{{\rm{h}}}}}}}^{-1})=\frac{{{{{{{\rm{n}}}}}}}_{{{{{{\rm{hydrobenzoin}}}}}}}}{{{{{{\rm{S}}}}}}\times {{{{{\rm{t}}}}}}}\times 100\%$$

The ${{{{{{\rm{n}}}}}}}_{{{{{{\rm{benzaldehyde}}}}}}}$, ${{{{{{\rm{n}}}}}}}_{{{{{{\rm{benzyl}}}}}}\,{{{{{\rm{alcohol}}}}}}}$, ${{{{{{\rm{n}}}}}}}_{{{{{{\rm{hydrobenzoin}}}}}}}$ represents the mole amounts of benzaldehyde, benzyl alcohol, and hydrobenzoin after the electrolysis. The ${{{{{{\rm{n}}}}}}}_{{{{{{\rm{benzaldehyde}}}}}}}^{0}$ represents the mole amount of benzaldehyde before reaction. Q refers to the total charge. F is Faraday’s constant. M is the catalyst loading. S is the catalyst area. t is the reaction time. The subscript represents the indicated chemical. The catalyst loading is determined by ICP-OES, which is ~0.34 mg cm^−2^ depending of the composite of the electrode material. Control experiments were carried out by changing the substrates in the catholyte with Pd-5s/Cu as the working electrode. Correspondence of the control experiment and the catholyte can be found in Supplementary Table S3.

### Electrochemical measurements

All the electrochemical measurements were carried out in the sample H-cell and potentiostat as for the activity evaluation. Linear scan voltammetry (LSV) curves were collected with a scan rate of 50 mV s^−1^ and stirring at ~900 rpm. Before the cyclic voltammetry (CV) test, electrocatalyst was scraped off from 1 cm^2^ of the electrode and dispersed in 0.1 mL of Nafion ethanol solution (5 wt%, 20 μL) under ultrasonication. Then, 60 uL of the prepared solution was dropped on glassy carbon electrodes (with geometric area of 0.5 cm^2^) and dried for the CV test. The CV curves were performed with a scan rate of 10 mV s^−1^ without stirring to avoid fluctuation. The electrochemically active surface area (ECSA) was determined by the double-layer capacitance (C_DL_) of the prepared electrocatalysts in 0.1 M KOH electrolyte under Ar atmosphere. There was no stirring during the ECSA measurements to minimize the fluctuation in the voltammogram. The scan rate ranged from 20 to 100 mV s^−1^. The detected current was plotted as a function of scan rate to obtain the C_DL_.

## Supplementary information

Supplementary Information

## Data Availability

All data generated in this study are included in the published article and its Supplementary Information. Source data are provided with this paper.

## Source data

Source Data
