# Supplementary material for: Electroreductive coupling of benzaldehyde by balancing the formation and dimerization of the ketyl intermediate
Source: Nat Commun. 2022 Dec 23;13:7909. doi: 10.1038/s41467-022-35463-3 (PMC9789095; doi:10.1038/s41467-022-35463-3)
Supplement: Supplementary file 1 — Supplementary Information [file 41467_2022_35463_MOESM1_ESM.pdf]

Supplementary Information

Electroreductive Coupling of Benzaldehyde by Balancing the Formation and Dimerization of the Ketyl Intermediate

Jia Yu[1,2‡], Peng Zhang[1,2‡], Lulu Li[1,2], Kailang Li[1,2], Gong Zhang[1,2], Jia Liu[1,2], Tuo Wang[1,2,3,4], Zhi-Jian Zhao[1,2], and Jinlong Gong[1,2,3]*

[1] *School of Chemical Engineering and Technology; Key Laboratory for Green Chemical Technology of Ministry of Education, Tianjin University, Tianjin 300072, China*

[2] *Collaborative Innovation Center of Chemical Science and Engineering (Tianjin), Tianjin 300072, China*

[3] *Haihe Laboratory of Sustainable Chemical Transformations, Tianjin 300192, China*

[4] *Joint School of National University of Singapore and Tianjin University, International Campus of Tianjin University, Binhai New City, Fuzhou 350207, China*

‡These authors contributed equally to this work.

*Corresponding author: jlgong@tju.edu.cn

Contents

1. Supplementary Methods

2. Supplementary Figures and Tables

3. Supplementary References

Supplementary Methods

Materials

Cu and Pd target (99.999%) were supplied by Zhongnuo Advanced Material Technology Co., Ltd, China. $HAuCl_4 \cdot 3H_2O$ (99%), NaOH (99%) and NH_4F solution (40%) were purchased from Aladdin Industrial Co. Ltd. H_2SO_4 (95-98%) was purchased from Sinopharm Chemical Reagent Co. Ltd. $Na_2S_2O_3$ (99.5%), isopropanol (HPLC), and HF (40.0%) were purchased from J&K Scientific Ltd. Methanol (HPLC), NH_4Cl (99.5%), HNO_3 (65%), and HCl (36%) were purchased from Tianjin Yuanli Technology Development Co. Ltd. (China). Potassium hydroxide (90%), benzyl alcohol (99%), hydrobenzoin (98%), 5,5-Dimethyl-1-pyrroline-N-oxide (DMPO, 98%+) and benzaldehyde (99%) were purchased from Maclean Biochemical Technology Co. Ltd. The carbon paper (CP, AvCarb GDS3250) was purchased from Xima Laya Photo-Electric Technology Co., Ltd. All aqueous solutions used ultrapure water (18.25 MΩ·cm) as the solvent. All gases used in this paper were from Air Liquide Co. Ltd., and all reagents were used directly without further purification.

Characterization

The field emission scanning electron microscope (FE-SEM, Regulus 8100) was employed to characterize the morphology of these Pd/Cu catalysts. Transmission electron microscope (TEM, JEOL JEM-200F, 200 kV) and High-angle annular dark-field scanning transmission electron microscopy (HAADFSTEM) were adopted to ensure the Pd distribution on Cu. X-ray powder diffraction (XRD; Rigaku, Smart, 9 kW Cu Kα radiation source working at 45 kV and 200 mA) and X-ray photoelectron spectroscopy with an Al Kα X-ray source (XPS, EscaLab MK) were applied to characterize the structure of prepared Pd/Cu catalysts.

In-Situ Attenuated Total Reflectance-Surface-Enhanced Infrared Absorption Spectroscopy (ATR-SEIRAS)

Preparation of Au plating solution: 3 g of NaOH was dissolved in 3 mL of 0.1 g mL^{-1} $HAuCl_4$ solution, and the color of the solvent turned from orange to orange-red upon NaOH dissolution. Meanwhile, 0.1337 g of NH_4Cl, 0.6205 g of $Na_2S_2O_3 \cdot 5H_2O$, and 0.9653 g of Na_2SO_3 were dissolved in 3 mL of water. Then, the two solutions were mixed and standing overnight for further use.

Au film deposition: The Au film was deposited on Si prism according to the method reported by Miyake et al.[1] All Si prisms were soaked in aqua regia overnight and then polished with Al_2O_3 (0.05 μm) until their surface turned hydrophobic. Afterward, the Si prisms were washed several times to clean the residual Al_2O_3. Before deposition, these cleaned Si was immersed in 40% NH_4F solution for 90 s to remove the surface oxides and afford an H-terminated surface. During the Au deposition process, the Si prism was transformed into a mixed solution containing 3 mL of Au platting solution and 1 mL of 2% HF at 55 ºC. Finally, the obtained Au film was washed with deionized water and dried for further use. These Pd/Cu catalysts were sputtered on Au film for subsequent ATR-SEIRAS test.

In-situ ATR-SEIRAS test: Before in-situ ATR-SEIRAS test, the reactor was washed with 1.0 M hydrochloric acid solution (HCl) and ethanol several times to remove any possible contaminants. The reactor was separated with a bipolar membrane. The Au film loaded with catalysts was used as the working electrode, using Ag/AgCl soaked in saturated KCl and glass carbon electrode as the reference and counter electrode, respectively. All spectra were recorded on Nicolet iS50 FT-IR spectrometer, with a resolution of 4 cm^{-1}. The background was collected in an argon-saturated 0.1 M KOH with 40 mmol L^{-1} benzaldehyde solution before each potential. All electrochemical experiments were recorded with a potentiostat (CompactStat.e20250, IVIUM).

In-situ Raman Spectroscopy Measurements

In-situ Raman experiments were carried out in a homemade electrolyzer using 0.1 M KOH with 40 mmol L^{-1} benzaldehyde as the electrolyte. The in-situ Raman samples were prepared on Cu foil. The electrode area was fixed to 1 cm^2. The electrochemical tests were conducted using a potentiostat Compact-Stat.e20250, IVIUM). The Raman tests were performed on a Confocal Raman Microscopy (RENISHAW, inVia reflex) equipped with a 633 nm He-Ne laser. The signal acquisition time is 90 s for each Raman spectrum.

Electron paramagnetic resonance (EPR) experiments.

In a typical EPR experiment, DMPO (75 mmol L^{-1}) was dissolved in 0.1 M KOH with 40 mmol L^{-1} benzaldehyde as the electrolyte. The electrolysis was carried out in a H-Cell for 8 min (−0.40 V *vs.* RHE), the sample was taken by capillary tubes for EPR experiments. The stirring rate was ~900 rpm during the electrolysis. The electrolyte was refreshed after each electrolysis. The electrolyte analyzed without electrolysis was also stirred for 8 min.

Density Functional Theory (DFT) Methods

Vienna ab initio simulation package (VASP) was employed to carry out calculations with the BEEF-vdW exchange-correlation functional.[2] The cut-off energy is 400 eV. At least 15 Å spacing was used to minimize periodic interactions between repeated slabs. The interactions between the atomic cores and electrons were described by the projector augmented wave (PAW) method. All structures were optimized until the force on each atom has been less than 0.02 eV/Å. To test the effect of the presence of liquid phase and potential on the reaction, the implicit solvent model was performed by the VASPsol code[3-5], which describes the effect of electrostatics, cavitation, and dispersion on the solute and solvent interaction by the linearized Poisson-Boltzmann equation. This method provides a relatively accurate approach to determine solvation energies of molecular and extended models. In details, one e^- charge was added and homogeneous background charge was assumed to simulate the reduction conditions. The results of implicit solvation and vacuum model are shown in Table S2. The difference in the values of binding and reaction energies is quite small and the reaction trends are consistent. The hydrogenation and dimerization of benzaldehyde was accessed on the Cu(111) and Pd(111) models. On the one hand, a five-layer Cu(111)-(4×4) slab with a (3×3×1) k-point grid and a five-layer Pd(111)-(4×4) slab with a (3×3×1) k-point grid were employed as models for the hydrogenation of benzaldehyde. On the other hand, a five-layer Cu(111)-(6×6) slab with a (1×1×1) k-point grid and a five-layer Pd(111)-(6×6) slab with a (1×1×1) k-point grid were used for the dimerization of benzaldehyde after hydrogenation. Three bottom layers were fixed while the upper layers were relaxed in these models.

The binding energy of the benzaldehyde (E_{ads}) was calculated as follows:

$$E_{ads} = E_{total} - E_{benzaldehyde} - E_{surface} \quad (1)$$

Supplemental Figures and Tables

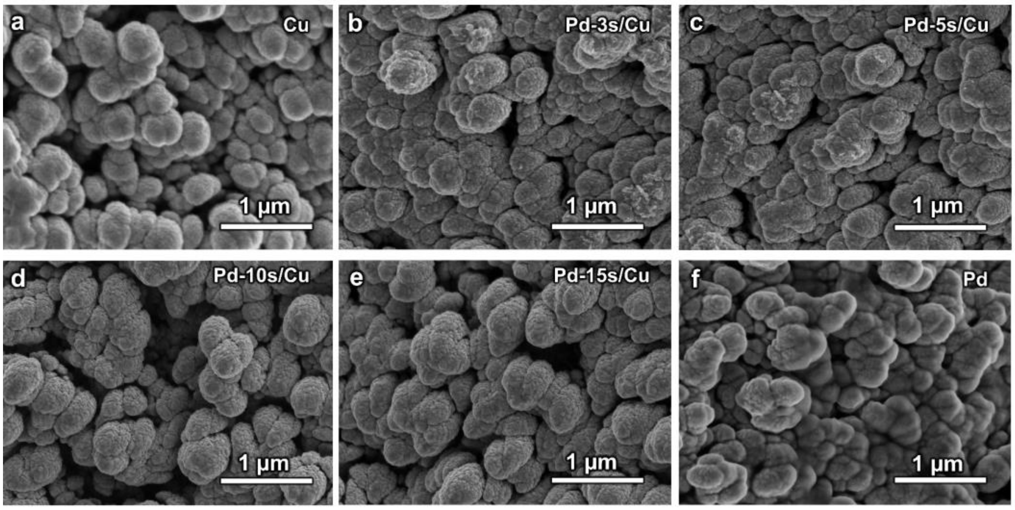

Supplementary Fig. 1. SEM images of different samples. a Cu, **b** Pd-3s/Cu, **c** Pd-5s/Cu, **d** Pd-10s/Cu, **e** Pd-15s/Cu, and **f** Pd.

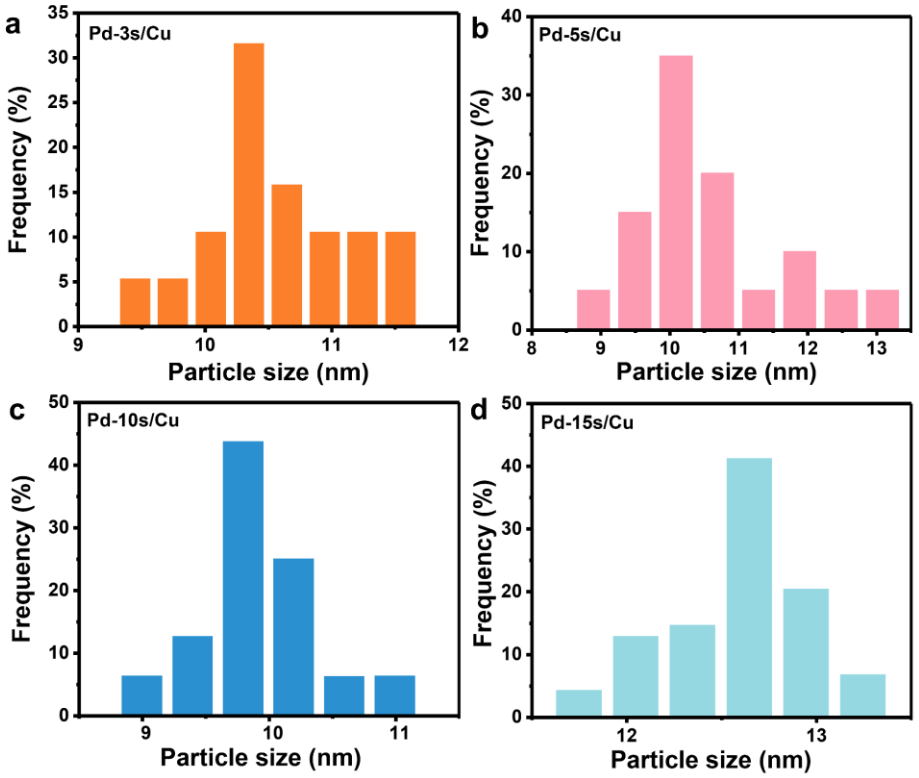

Supplementary Fig. 2. Size distributions of Pd particles in different samples. a Pd-3s/Cu, **b** Pd-5s/Cu, **c** Pd-10s/Cu, and **d** Pd-15s/Cu.

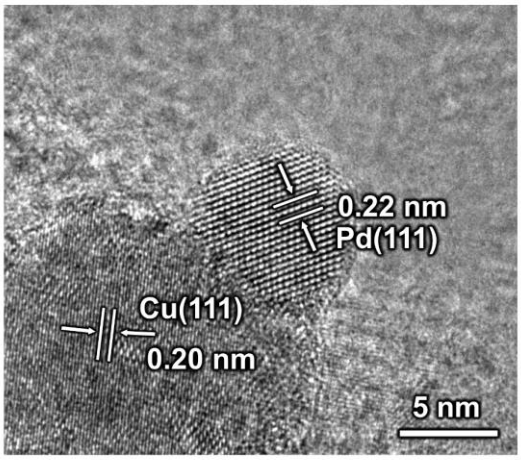

Supplementary Fig. 3. High-resolution TEM (HRTEM) image. The HRTEM of Pd-5s/Cu.

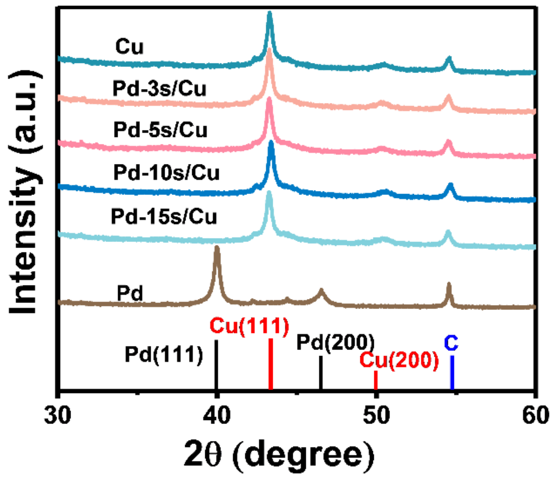

Supplementary Fig. 4. XRD patterns of prepared catalysts. The XRD patterns of Cu, Pd-3s/Cu, Pd-5s/Cu, Pd-10s/Cu, Pd-15s/Cu and Pd.

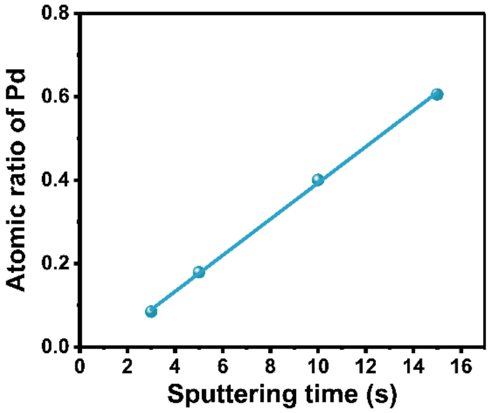

Supplementary Fig. 5. Atomic ratio of Pd. The atomic ratio of Pd showed a linear relationship with its sputtering time.

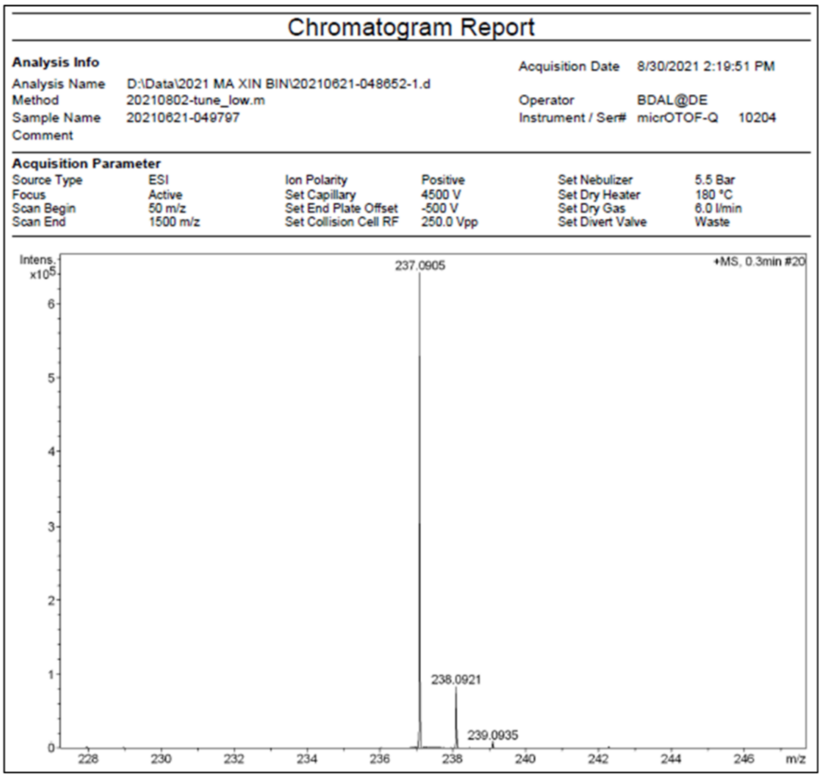

Supplementary Fig. 6. High resolution mass spectrum. The high resolution mass spectrum of hydrobenzoin.

The electrolyte was diluted with CH_3OH and the benzaldehyde and benzyl alcohol were not detected for low boiling point compared with hydrobenzoin.

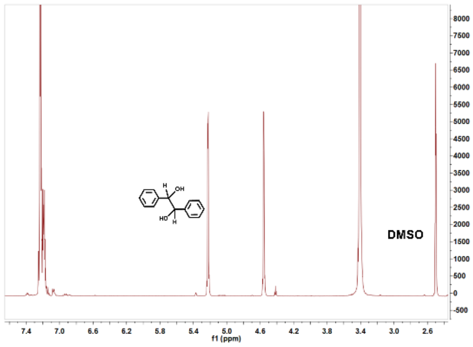

Supplementary Fig. 7. ^{1}H Nuclear magnetic spectrum. The ^{1}H Nuclear magnetic spectrum of hydrobenzoin.

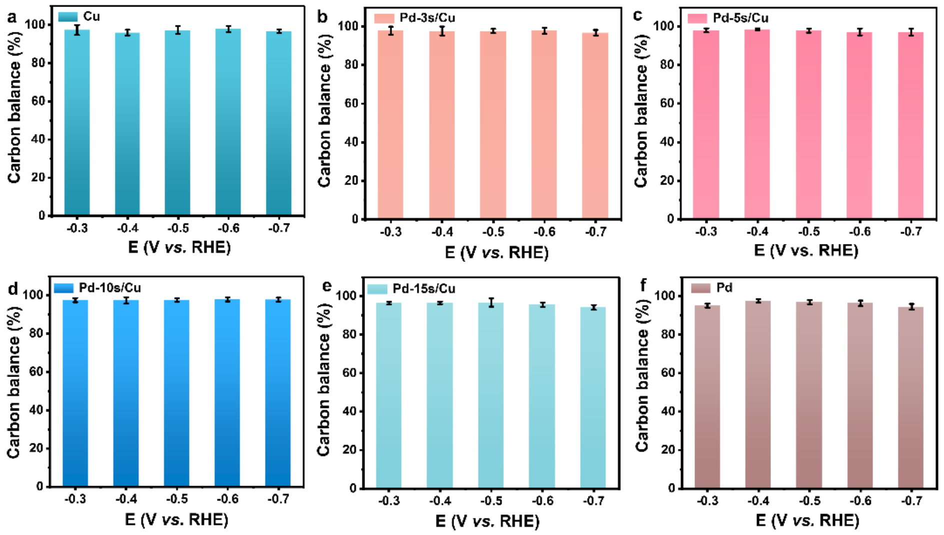

Supplementary Fig. 8. Carbon balance of benzaldehyde electrolysis over different samples. **a** Cu, **b** Pd-3s/Cu, **c** Pd-5s/Cu, **d** Pd-10s/Cu, **e** Pd-15s/Cu, and **f** Pd. Error bars represent the standard deviation from at least three independent measurements.

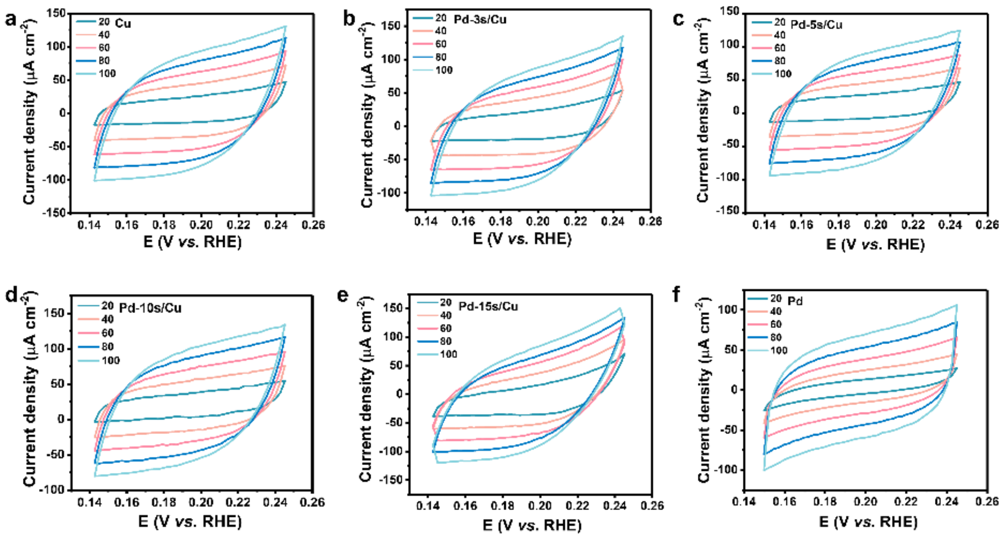

Supplementary Fig. 9. The non-Faraday CV profiles obtained for different samples. **a** Cu, **b** Pd-3s/Cu, **c** Pd-5s/Cu, **d** Pd-10s/Cu, **e** Pd-15s/Cu, **f** Pd. Sweeping rates are 20, 40, 60, 80, and 100 mV s^{-1}.

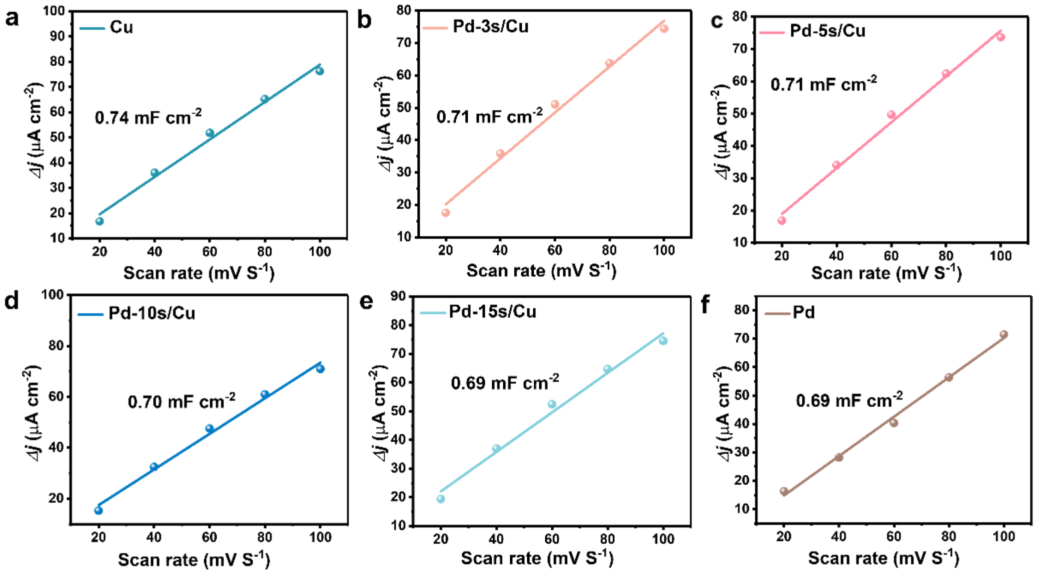

Supplementary Fig. 10. Double-layer capacitance measurements of different samples. a Cu, **b** Pd-3s/Cu, **c** Pd-5s/Cu, **d** Pd-10s/Cu, **e** Pd-15s/Cu, and **f** Pd.

The surface roughness factor of Cu and Pd with a C_{dl} nearly 0.03 mF cm^{-2} is regard as 1[6, 7]. Thus, the surface roughness factor was 24.6 (Cu), 23.7 (Pd-3s/Cu, Pd-5s/Cu), 23.3 (Pd-10s/Cu), 23.0 (Pd-15s/Cu, Pd), respectively.

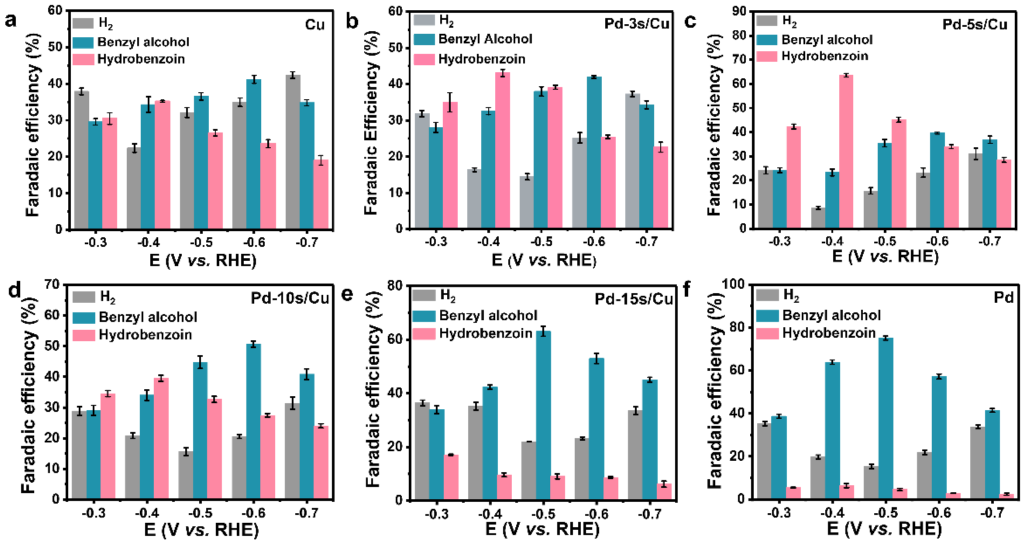

Supplementary Fig. 11. Faradaic efficiencies of benzaldehyde reduction products for different samples. a Cu, **b** Pd-3s/Cu, **c** Pd-5s/Cu, **d** Pd-10s/Cu, **e** Pd-15s/Cu, and **f** Pd. Error bars represent the standard deviation from at least three independent measurements.

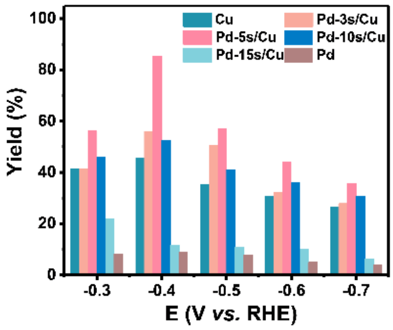

Supplementary Fig. 12. Yield of hydrobenzoin. The yield of hydrobenzoin for different catalysts at varied potentials after accumulated 100 C.

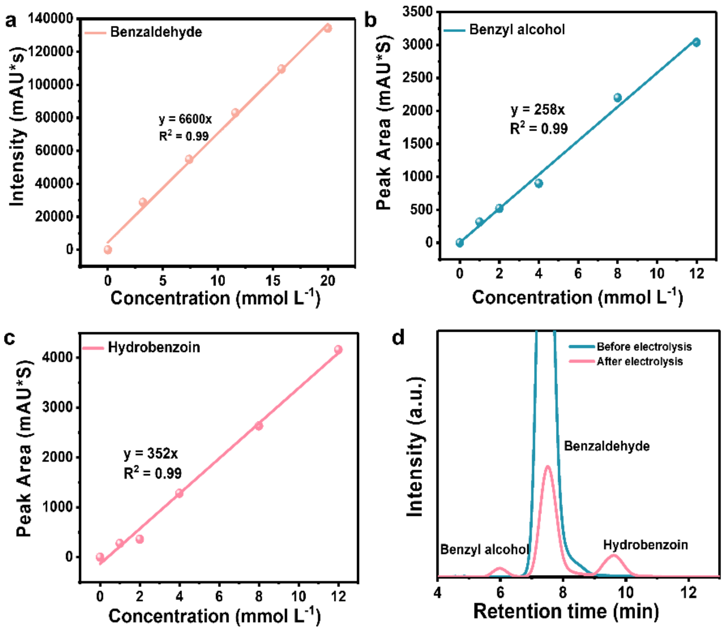

Supplementary Fig. 13. Calibration curve and high performance liquid chromatography (HPLC) spectra. a Calibration curve of benzaldehyde, **b** benzyl alcohol, **c** hydrobenzoin. **d** HPLC spectra of Pd-5s/Cu.

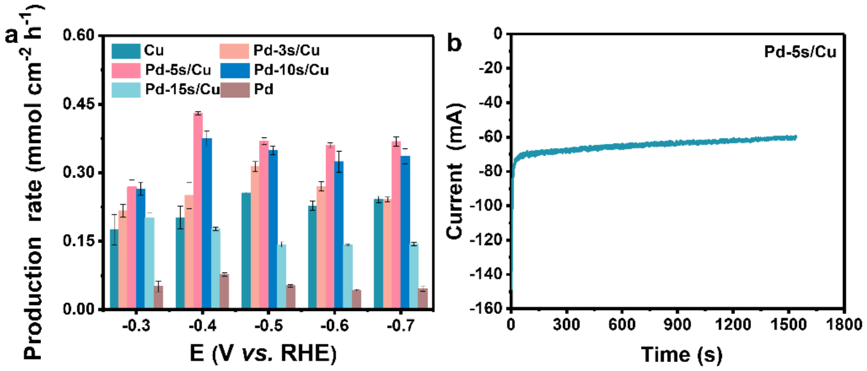

Supplementary Fig. 14. Production rate and I-t curves. a Production rate (mmol cm^{-2} h^{-1}). **b** I-t curves on Pd-5s/Cu at -0.40 V *vs.* RHE. Error bars represent the standard deviation from at least three independent measurements.

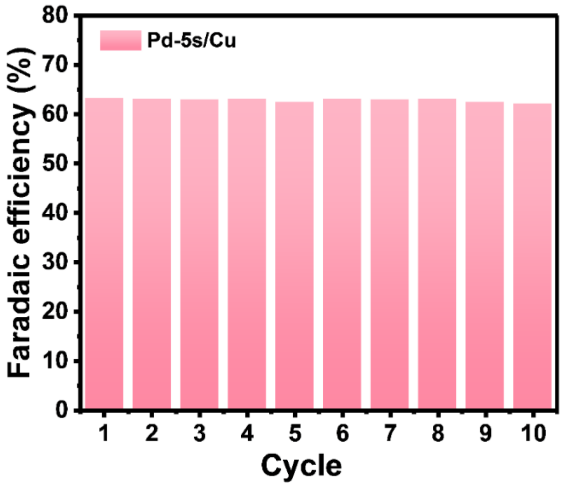

Supplementary Fig. 15. Stability tests. The Faradaic efficiencies of hydrobenzoin for Pd-5s/Cu in stability tests.

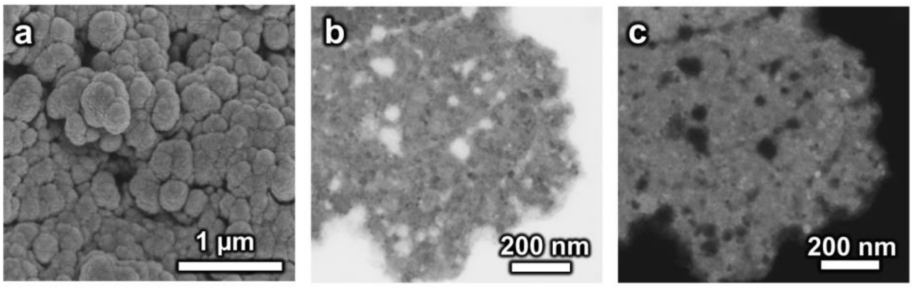

Supplementary Fig. 16. Structural characterization of Pd-5s/Cu after stability tests. a SEM, **b** TEM, and **c** HAADFSTEM images of Pd-5s/Cu after stability tests.

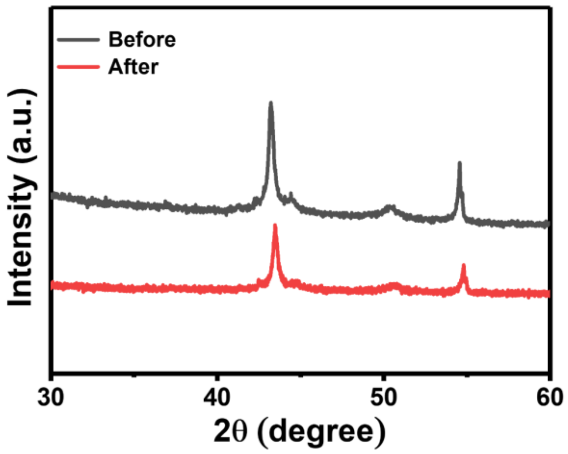

Supplementary Fig. 17. XRD patterns before and after the stability tests. The XRD patterns of Pd-5s/Cu before and after the stability tests.

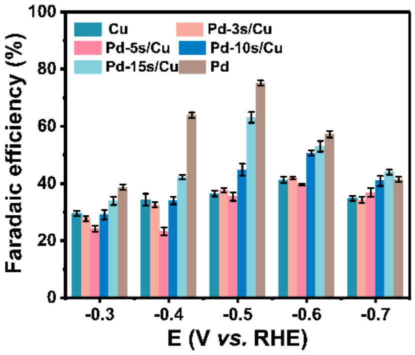

Supplementary Fig. 18. Faradic efficiencies of benzyl alcohol. The Faradic efficiencies of benzyl alcohol on Cu, Pd-3s/Cu, Pd-5s/Cu, Pd-10s/Cu, Pd-15s/Cu and Pd at different applied potentials. Error bars represent the standard deviation from at least three independent measurements.

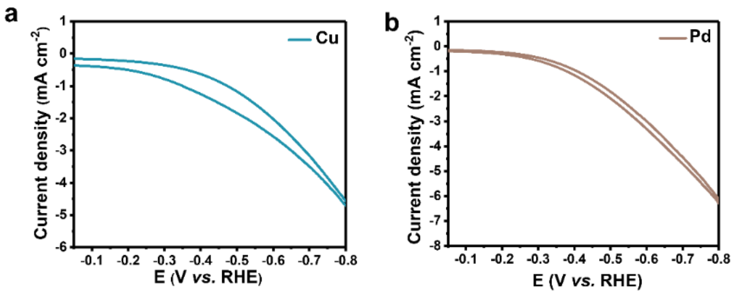

Supplementary Fig. 19. Cyclic voltammograms of different samples on a glassy carbon electrode. a Cu and **b** Pd in 0.1 M KOH solution.

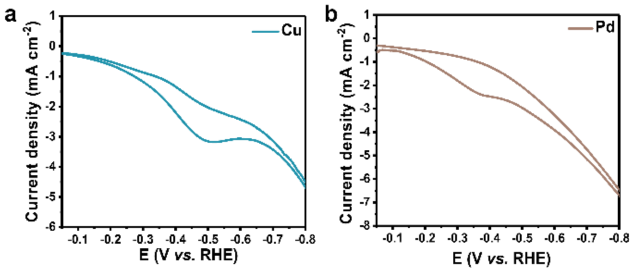

Supplementary Fig. 20. Cyclic voltammograms of different samples on a glassy carbon electrode. a Cu and **b** Pd in 0.1 M KOH solution containing 40 mmol L^{-1} benzaldehyde.

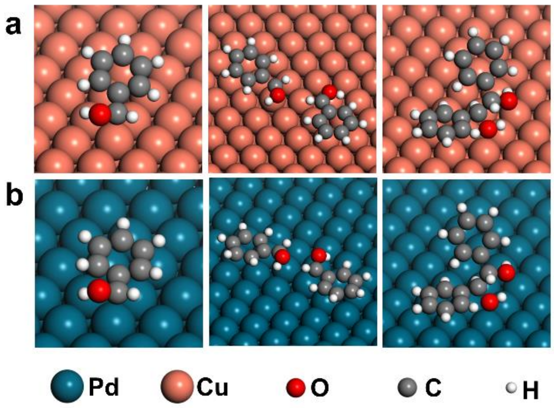

Supplementary Fig. 21. The front view of the optimized states for dimerization process. The front view of dimerization process on **a** Cu and **b** Pd.

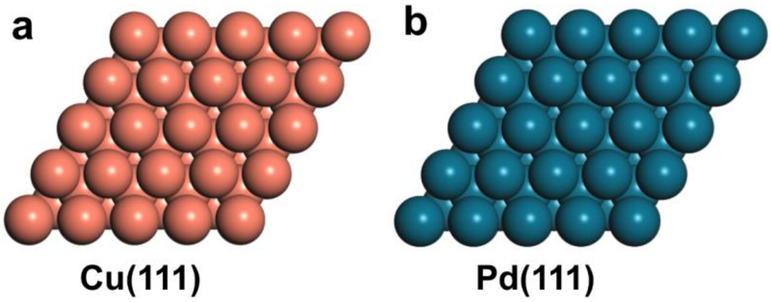

Supplementary Fig. 22. DFT calculation models. a Cu(111) and **b** Pd(111).

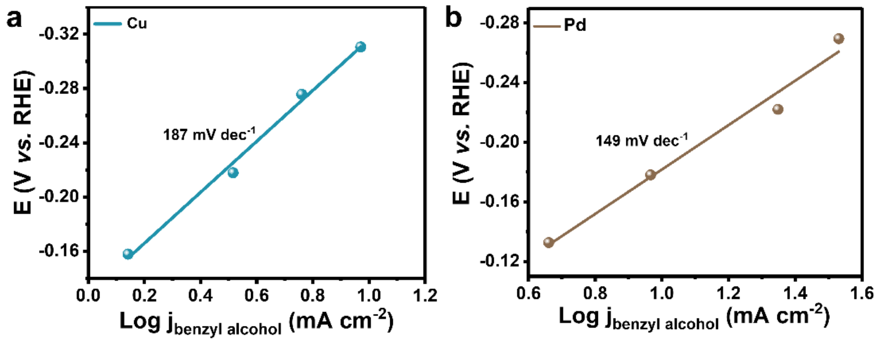

Supplementary Fig. 23. Tafel plots of different samples. a Cu. **b** Pd. Calculated from the partial current density of benzyl alcohol at different applied potentials. E (Real potential) = E (Applied potential) – I (Current) × R_u, where the R_u is the resistance for charge transfer obtained by electrochemical impedes measurements.

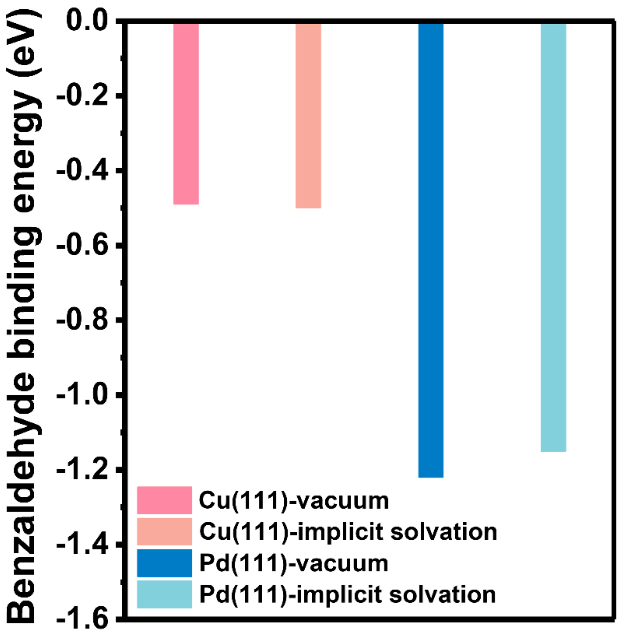

Supplementary Fig. 24. Binding energy of benzaldehyde. Binding energy of benzaldehyde on Cu(111) and Pd(111) in vacuum and implicit solvation conditions.

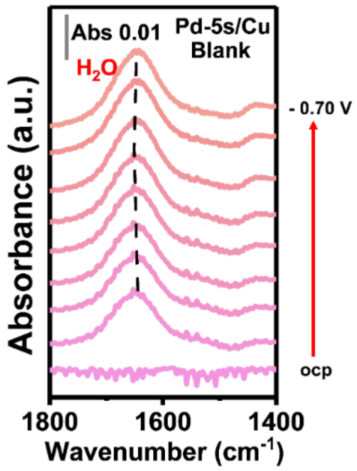

Supplementary Fig. 25. In-situ ATR-SEIRAS investigation in 0.1 M KOH. In-situ ATR-SEIRAS investigation of Pd-5s/Cu in 0.1 M KOH without benzaldehyde.

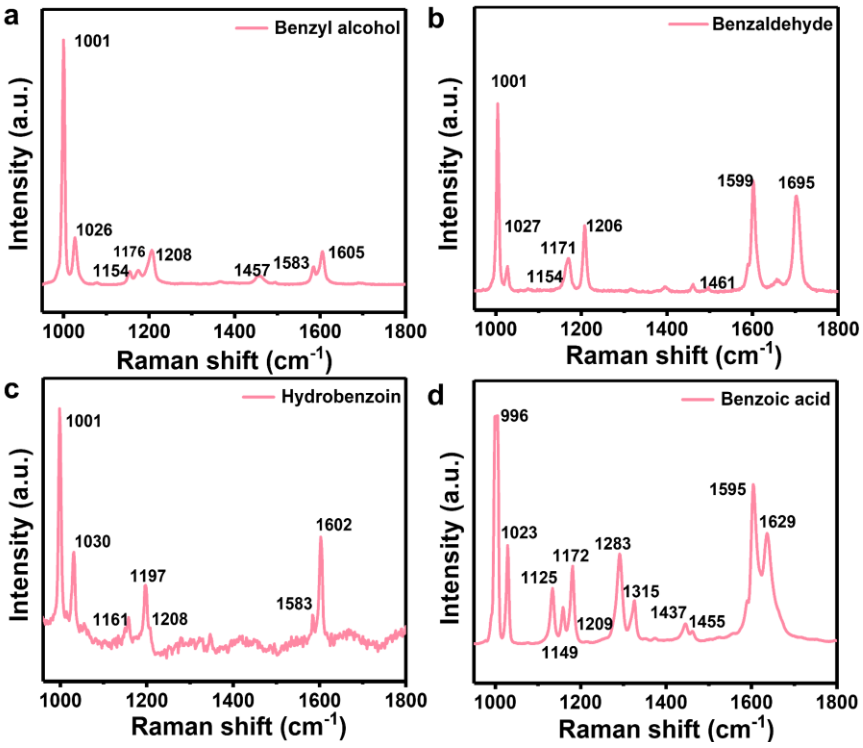

Supplementary Fig. 26. Raman spectra of pure chemicals. a benzaldehyde, **b** benzyl alcohol, **c** hydrobenzoin, and **d** benzoic acid.

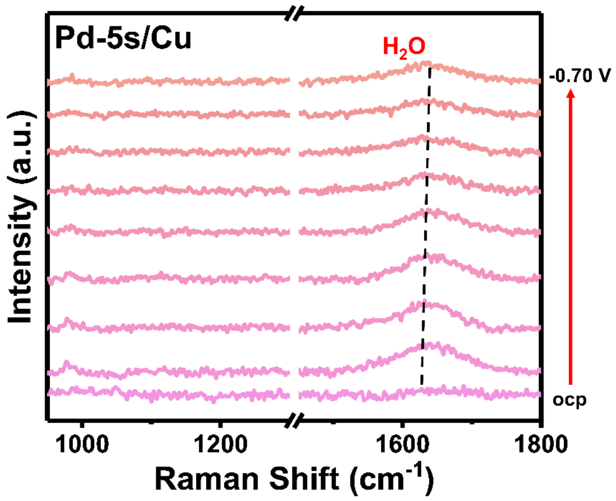

Supplementary Fig. 27. Raman spectra in 0.1 M KOH. The Raman spectra of Pd-5s/Cu in 0.1 M KOH without benzaldehyde at different applied potentials.

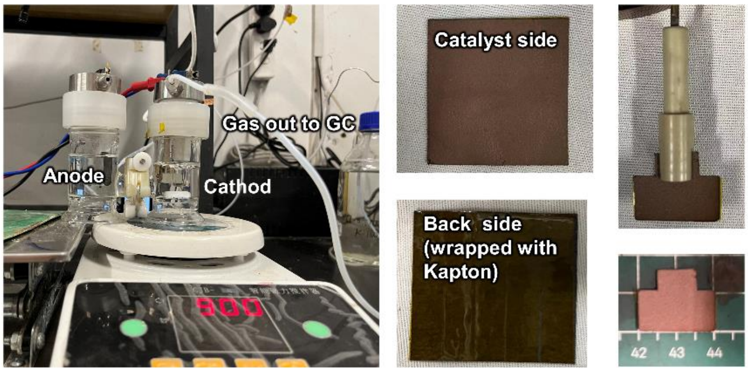

Supplementary Fig. 28. Schematic diagram of activity evaluation setup. The H-type cell and the working electrode used in the experiment.

Supplementary Table 1.

The benzaldehyde reduction performance of recent works

**Catalyst**	**Approaches**	**FE (%)**	**Yield (%)**	**Production rate**	**Ref**
$Ni/ZnIn_2S_4$	Photocatalysis	/	98.1	1.00 mmol mg^{-1} h^{-1}	8
CdS/SiO_2	Photocatalysis	/	84.5	0.02 mmol mg^{-1} h^{-1}	9
CdS QDs	Photocatalysis	/	68	/	10
CdS NPs	Photocatalysis	/	10	0.138×10^{-3} mmol mg^{-1} h^{-1}	11
Carbon felt	Electrocatalysis	/	/	1.50×10^{-3} mmol mg^{-1} h^{-1}	12
Carbon paper	Electrocatalysis	99	99	0.6 mmol cm^{-2} h^{-1}	13
Commercial Cu foil	Electrocatalysis	37	/	/	14
Pd/Cu	Electrocatalysis	63.2	85.3	1.27 mmol mg^{-1} h^{-1}<br>0.43 mmol cm^{-2} h^{-1}	**This work**

Supplementary Table 2.

Comparison of implicit solvation and vacuum model

Model	Binding Energy of Benzaldehyde (eV)	Reaction energy of $*C_6H_5CHO+*H$ to $*C_6H_5CHOH$ (eV)	Reaction energy of $2*C_6H_5CHOH$ to $*(C_6H_5CHOH)_2$ (eV)
Pd(111) implicit	−1.15	0.03	−1.22
Pd(111) vacuum	−1.22	−0.18	0.98
Cu(111) implicit	−0.50	0.57	−1.07
Cu(111) vacuum	−0.49	0.5	−0.89

Supplementary Table 3.

The catholyte composition of the control experiments

**Name**	**Control experiment**	**Catholyte**
Control 1	Benzyl alcohol reduction	40 mmol L^{-1} benzyl alcohol in 0. 1 M KOH
Control 2	Benzaldehyde reduction	40 mmol L^{-1} benzaldehyde in 0. 1 M KOH
Control 3	Benzyl alcohol and benzaldehyde co-reduction	40 mmol L^{-1} benzyl alcohol + 40 mmol L^{-1} benzaldehyde in 0. 1 M KOH
Control 4	Hydrobenzoin reduction	0.5 mL of 100 mg mL^{-1} hydrobenzoin methanol solution added in 0.1 M KOH

Supplementary References

1. Miyake, H., Ye S., Osawa M. Electroless deposition of gold thin films on silicon for surface-enhanced infrared spectroelectrochemistry. *Electrochem. Commun.* **4**, 973-977 (2002).
2. Kresse, G., Furthmuller J. Efficient iterative schemes for ab initio total-energy calculations using a plane-wave basis set. *Phys. Rev. B. Condens. Matter.* **54**, 11169-11186 (1996).
3. Mathew, K., *et al.* Implicit self-consistent electrolyte model in plane-wave density-functional theory. *J. Chem. Phys.* **151**, 234101 (2019).
4. Mathew, K., *et al.* Implicit solvation model for density-functional study of nanocrystal surfaces and reaction pathways. *J. Chem. Phys.* **140**, 084106 (2014).
5. Lopez-Ruiz, J. A., *et al.* Understanding the role of metal and molecular structure on the electrocatalytic hydrogenation of oxygenated organic compounds. *ACS Catal.* **9**, 9964-9972 (2019).
6. Gubanova, E., *et al.* Structure-Dependent Electrical Double-Layer Capacitances of the Basal Plane Pd(hkl) Electrodes in $HClO_4$. *The Journal of Physical Chemistry C* **126**, 11414-11420 (2022).
7. Zhang, G., *et al.* Efficient CO_2 electroreduction on facet-selective copper films with high conversion rate. *Nat Commun* **12**, 5745 (2021).
8. Han, G., Liu X., Cao Z., Sun Y. Photocatalytic pinacol C-C coupling and jet fuel precursor production on $ZnIn_2S_4$ nanosheets. *ACS Catal.* **10**, 9346-9355 (2020).
9. Qi, M.-Y., *et al.* Efficient photoredox-mediated C-C coupling organic synthesis and hydrogen production over engineered semiconductor quantum dots. *ACS Catal.* **10**, 14327-14335 (2020).
10. McClelland, K. P., Weiss E. A. Selective photocatalytic oxidation of benzyl alcohol to benzaldehyde or C-C coupled products by visible-light-absorbing quantum dots. *ACS Appl. Energy Mater.* **2**, 92-96 (2018).
11. Mitkina, T., *et al.* Visible light mediated homo- and heterocoupling of benzyl alcohols and benzyl amines on polycrystalline cadmium sulfide. *Org. Biomol. Chem.* **10**, 3556-3561 (2012).
12. Andrews, E., *et al.* Performance of base and noble metals for electrocatalytic hydrogenation of bio-oil-derived oxygenated compounds. *ACS Sustain. Chem. Eng.* **8**, 4407-4418 (2020).
13. Liu, C. B., *et al.* Selectivity origin of organic electrosynthesis controlled by electrode materials: A case study on pinacols. *ACS Catal.* **11**, 8958-8967 (2021).
14. Anibal, J., Malkani A., Xu B. J. Stability of the ketyl radical as a descriptor in the electrochemical coupling of benzaldehyde. *Catal. Sci. Technol.* **10**, 3181-3194 (2020).
